# Nanobiotechnology today: focus on nanoparticles

**DOI:** 10.1186/1477-3155-5-11

**Published:** 2007-12-30

**Authors:** Mikhail Soloviev

**Affiliations:** 1 Royal Holloway University of London, Egham, Surrey, TW20 0EX, UK

## Abstract

In the recent years the nanobiotechnology field and the Journal of Nanobiotechnology readership have witnessed an increase in interest towards the nanoparticles and their biological effects and applications. These include bottom-up and molecular self-assembly, biological effects of naked nanoparticles and nano-safety, drug encapsulation and nanotherapeutics, and novel nanoparticles for use in microscopy, imaging and diagnostics. This review highlights recent Journal of Nanobiotechnology publications in some of these areas .

## Bottom-up nanobiotechnology and biology-inspired nanoparticles

Some of the most promising applications of biologically inspired nanoparticles have so far been in nanobiotechnology and in tissue- and cell-specific drug delivery in particular. Unlike liposomes, dendrimers, metal and semiconductor nanoparticles, the nanoparticles made of biopolymers, such as bacterial spores, viruses and alike are naturally uniform in size and offer precise control for the surface-displayed targeting groups and their components. Furthermore, such biological nanoparticles may be produced recombinantly relatively easy and at low cost, and their assembly may be easily followed using a plethora of molecular and cellular approaches and instrumental techniques. The stability of nanoparticles made of biopolymers is one of the limiting factors which will determine the range of their applications. In their most recent paper *Caldeira and Peabody *[1] investigated in vitro assembly and stability of virus-like icosahedral capsid of the RNA bacteriophage PP7 particles. Contrary to expectations, recombinant fusion of the subunits have not stabilized PP7 virus-like particles against thermal denaturation, whilst disulphide bonds between coat protein dimers greatly increased the viral particles' stability.

A loading capacity of nanoparticles made of biopolymers is another important criterion in the use of such nanoparticles as nano-containers for specific targeting applications. A comprehensive study of the assembly and stability of canine parvovirus-like particles (CPV) was conducted by *Gilbert et al *[2] who employed a novel strategy, based on Fluorescence Correlation Spectroscopy analysis, to monitor the assembly of a series of truncated monomeric canine parvovirus VP2 structural proteins and their GFP fusions. The trancations ranged from 0 (native protein) to 40 amino acids. Intriguingly, only one truncated variant (-14 amino acids) failed to assemble into a CPV-like particle, which was confirmed independently using more traditional confocal and electron microscopy approaches. The GFP "load" did not prevent nanoparticle assembly.

The ability to manipulate and direct CPV assembly is of critical importance in the field of target-specific drug delivery. Because CPV has naturally high affinity to transferrin receptors (TfRs), which are often over-expressed on tumor cells, CPV might be used for specific targeting of tumour cells directly. *Singh et al *[3] have utilized this ability of CPVs and studied targeting of HeLa, HT-29 and MDA-MB231 cells and the internalization of native and modified ("loaded") CPVs. The assembled CPV-like nanoparticles were found to withstand conjugation with chemotherapeutic drugs, remain intact following their purification and internalise within 2 hours through TfRs receptors.

## Biological effects and therapeutic applications

Traditional strategies towards the tissue-specific drug delivery utilise cytotoxic drugs attached to targeting moieties (e.g. towards TfRs receptors mediating cell-specific targeting and internalisation). In their recent report *Mondalek et al *[4] have shown that nanoparticle internalisation can be enhanced by the use of an external magnetic field and targeted magnetic delivery. To illustrate this, a model similar to the human round window membrane has been developed and superparamagnetic iron oxide (Fe_3_O_4_) nanoparticles were magnetically transported through three co-cultured layers of cells. Such magnetic gradient-forced transport is minimally invasive, does not compromise epithelial confluence and has the potential to enhance the therapeutic benefits of magnetic nanoparticles-based drugs and reduce their toxicity.

Magnetic nanowires are another example of paramagnetic nanomaterial especially suitable for nanobiotechnology applications due to their size and anisotropy (unlike traditional anisotropic magnetic nanoparticles. *Prina-Mello and co-workers *have shown that Nickel nanowires, grown in alumina membranes, can be introduced into adherent and suspended cells and be used for cell manipulation, identification and separation [5]. The authors have also shown that internalised nanowires can be manipulated (re-oriented) whilst inside the cells without inducing any anisotropy in the population of adherent cells.

In addition to their ever more increasing use in molecular separations and targeting, magnetic nanoparticles were shown to also increase stability, activity and functionality of enzymes immobilised on the surface of the particles [6]. Kinetic studies of free and bound Cholesterol oxidase revealed structural and conformational changes of the immobilised enzyme which resulted in the reduction of activation energy upon binding onto iron oxide (Fe_3_O_4_) nanoparticles. The binding to nanoparticles further improved the storage stability of the enzyme, increased its tolerance to the variation in reaction pH and its thermal stability (increased twice at 60°C). The above effects were observed with particles ranging between 9.7 and 56.4 nm in size. Protein-nanoparticle interactions and the immobilisation kinetics onto L-aspartic acid-modified iron oxide (Fe_3_O_4_) nanoparticles and 3-mercaptopropionic acid-modified gold coated Fe_3_O_4_/Au nanoparticles has been reported by *Kouassi and Irudayaraj *previously [7].

In an independent study, *Mukherjee et al *reported that the immobilisation of anti-VEGF antibodies on gold nanoparticles increase the ability of these antibodies to induce apoptosis in Chronic Lymphocytic Leukemia B cells [8]. The induction of apoptosis with gold-conjugated anti-VEGF antibodies was significantly higher than the CLL cells exposed to antibodies alone or to unconjugated gold nanoparticles. The authors attribute the effect to the increased concentration of drug and improved intracellular delivery, although improvements in antibody stability, conformational changes and the nanoparticles' cytotoxic effect on the target cells cannot be discounted.

A complex character of the interactions of inorganic nanoparticles with viral particles has been investigated by *Elechiguerra et al *[9]. The authors unequivocally demonstrated size- and site-dependent interaction of silver nanoparticles with gp120 surface glycoproteins of the HIV-1 virus. The binding and the inhibition of virus binding to host cells is limited to particles ranging between 1 and 10 nm in size.

In contract to [8,9], *Williams et al *observed no effect on cell proliferation or any signs of toxicity when *Escherichia coli *were incubated with silica, silica/iron oxide, and gold nanoparticles [10]. Studying the interaction of inorganic nanoparticles with biological targets, whether molecules, viruses, bacteria, cells, tissues or organisms, as well as the nano-safety aspects and the long-term effects of that interaction might present a challenge to the scientific community due to the sheer number of materials, nanoparticle preparation methods and functionalization techniques. There is no universal "nanoparticle" to fit all the cases, and the multitude of "grey goo" scenarios, first hinted at by nanotechnology theorist Dr. K. Eric Drexler in his 1986 book "Engines of Creation", has raised hair on the heads of many safety officers and researchers active in the field, including Peter Hoet, Irene Brüske-Hohlfeld and Oleg Salata who's paper on the health risks associated with the nanoparticles remains the most cited paper in the Journal of Nanobiotechnology to date [11].

## Concluding remarks

This month the Journal of Nanobiotechnology celebrates 5 years since its creation. On behalf of the Editorial board I would like to thank all the authors for their precious work and excellent manuscripts, the reviewers for their invaluable service to the field and the Editorial Board and many Editors of other BioMed Central publications for their continuous support and encouragement. My special thanks go to the publisher, BioMed Central (London). I would like to invite the wider scientific community to join the fast growing readership of this Open Access Journal and also to consider it for publishing your own work. And finally, my warmest wishes to everybody for the coming Christmas and the New Year (with apologies to the followers of other calendars and religions). This New Year promises to be the warmest ever but we have yet to see a manuscript on the use of *Nano-*bio-technology for solving this truly *Planetary*-scale problem of global warming.
